# Impact of Interfering Substances on the Bactericidal Efficacy of Different Commercially Available Hypochlorous Acid-Based Wound Irrigation Solutions Commonly Found in South-East Asia

**DOI:** 10.3390/antibiotics13040309

**Published:** 2024-03-28

**Authors:** Jiann Wen Yap, Neni Iffanida Ismail, Cheng Shoou Lee, Ding Yuan Oh

**Affiliations:** 1 Wound and Stoma Care Unit, Queen Elizabeth Hospital, Karung Berkunci No. 2029, Kota Kinabalu 88586, Sabah, Malaysia; yapjiannwen@moh.gov.my; 2 TECOLAB SDN BHD, J-2-6, Pusat Komersial Jalan Kuching, Kuala Lumpur 51200, Malaysia; neni@tecolab-global.com (N.I.I.); marven@tecolab-global.com (C.S.L.); 3 Schülke & Mayr (Asia) Pte Ltd., 10 Jalan Kilang #04-01/02/03, Singapore 159410, Singapore

**Keywords:** commercial hypochlorous acid-based solutions, wound cleansing, antimicrobial activity, quantitative suspension method, antimicrobial, protein interference

## Abstract

The high prevalence of chronic wounds is a growing concern. Recently, hypochlorous acid (HOCl)-based solutions were introduced as an alternative antimicrobial for wound cleansing. In this study, we assessed the in vitro bactericidal activities of seven commercially available wound irrigation products commonly found in South-East Asia. The evaluation was conducted using quantitative suspension method, EN 13727 in either low or high protein conditions. Under low protein conditions, four out of the five HOCl products achieved bactericidal activity (≥5 log_10_ reduction factor; RF) within 2–5 min, and only one product achieved 5 log_10_ RF at 15 s. None of the HOCl achieved 5 log_10_ RF under high protein, even after 30 min of exposure time. In contrast, protein interference on the antimicrobial activities of polyhexamethylene biguanide-based product is less pronounced (low protein: 60 s vs. high protein: 2 min to attain ≥5 log_10_ RF). Octenidine dihydrochloride is the only active not affected by protein interference achieving ≥5 log_10_ RF within 15 s in both low and high protein conditions. These findings warrant the need to screen antimicrobial wound care products, especially HOCl-based products, in high protein condition to better reflect the antimicrobial activities in wound care.

## 1. Introduction

The growing prevalence of chronic wounds has become a global concern, with approximately 18.6 million people worldwide affected by a chronic wound such as diabetic foot ulcers (DFU) each year [1]. The growing number of people suffering from chronic wounds such DFU in South-East Asia is alarming [2]. The associated health–economic impact of chronic wounds in various countries are considerable, with 2–4% of the national health expenditure (e.g., Australia, the UK, Singapore, and Scandinavian countries) targeted solely on the care of chronic wounds alone [3]. Chronic wounds are commonly manifested with biofilms that result in delayed healing and recurring infection episodes [4]. Due to increasing antimicrobial resistance, antiseptics have played a significant role in wound infection management. According to the recently published “Consensus for the use of antiseptic in practice, antiseptic is generally considered the first choice when it comes to treating localized infected wound” [5]. 

One of the key selection criteria for choosing an antiseptic by clinicians is how well the antiseptic eliminate microbial contaminants [5]. To date, the screening of the antimicrobial efficacy of wound antiseptic still relies on in vitro testing methodology, such as the EN 13727 and ASTM E2783—a suspension testing system where antiseptic is tested against planktonic bacteria [6,7]. Being different from skin antisepsis, the application of antiseptic in wound care faces the additional challenge of a high protein environment [6]. It is well-established that protein can affect the antimicrobial efficacies of antimicrobial agents [8]. Although the exact mechanism remains to be elucidated, the preliminary understanding of these interference commonly known as ‘protein error’ is via chemical and/or ionic interactions [8,9,10]. To stimulate a realistic wound environment for testing, organic soiling, such as wound exudate extract from patient’s dressings, and the combination of different protein composition have been used to stimulate physiological wound environment [11,12,13]. Severing and colleagues (2022) recently demonstrated that a modified peptide challenge yielded comparable results to wound exudates when employed as an interfering substance in the EN 13727 test—establishing a simpler and more standardized method to stimulate a wound environment without the need for human wound exudate [12].

The act of wound cleansing is a critical step towards wound bed preparation, often involving the usage of an irrigation solution to rinse, soak, and/or irrigate [5,14]. Among the different antimicrobial irrigation solutions, hypochlorous acid (HOCl)-based solutions have increasingly been introduced as an alternative for wound care antisepsis. This is especially prominent in South-East Asia, where a wide range of HOCl-based irrigation solutions is available for use. HOCl-based irrigation solutions are chlorine-based and chlorine-releasing agents, where their antimicrobial effect relies on the interaction between the reactive chlorine species with microorganisms to achieve killing via oxidation [15,16]. It is known that not all products containing HOCl share similar antimicrobial efficacies with the latter heavily dependent on formulary constituents and chlorine concentration [12,14,15]. However, there is limited reporting on the antimicrobial efficacies, especially in the different protein testing conditions of the various HOCl-based irrigation solutions in South-East Asia. 

Using EN 13727:2012+A2:2015 as the testing methodology, this study aims to establish the antimicrobial efficacy of five commercially available HOCl-based solutions under the stimulated wound environment of high protein concentration. 

## 2. Results

### 2.1. Efficacy of Wound Irrigation Solutions under Low Protein Condition

**Figure 1:**
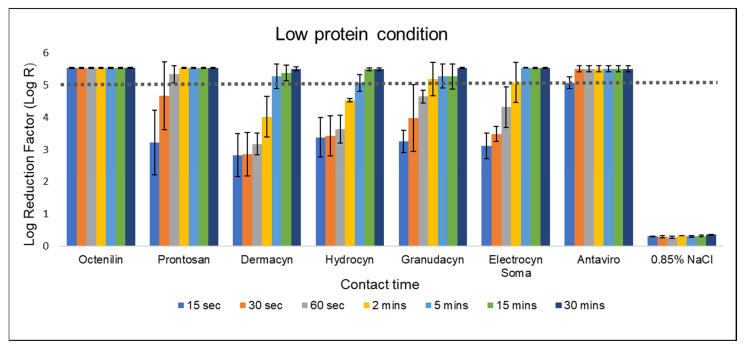
Bactericidal efficacy of various wound irrigation solutions against *S. aureus* according to EN 13727 in low protein condition. Log reduction achieved by the eight test solutions against *S. aureus* was evaluated under low protein condition, ranging from 15 s to 30 min of contact time. The dotted line represents the minimum requirement (logarithmic reduction factor of 5) to demonstrate bactericidal activity according to EN 13727. The bars represent mean values with standard deviations of two independent tests.

**Figure 2:**
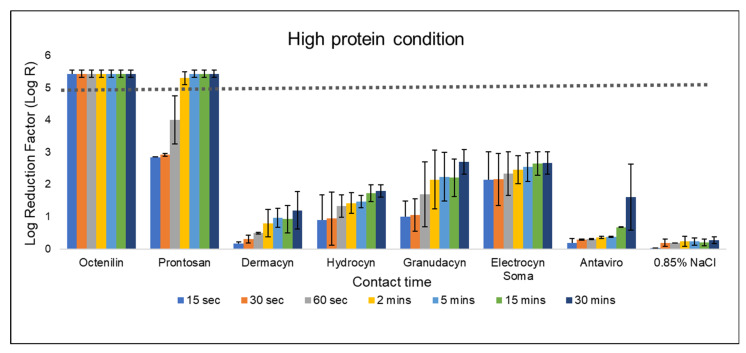
Bactericidal efficacy of various wound irrigation solutions against *S. aureus* according to EN 13727 in high protein condition. Log reduction achieved by the eight test solutions against *S. aureus* was evaluated under high protein condition, ranging from 15 s to 30 min of contact time. The dotted line represents the minimum requirement (logarithmic reduction factor of 5) to demonstrate bactericidal activity according to EN 13727. The bars represent mean values with standard deviations of two independent tests.

According to EN 13727, a test product is considered to possess bactericidal activity against specific bacteria when it achieves a 5-log reduction. In this study, 0.85% NaCl served as the negative control, and as expected, bacterial reduction was minimal to none (Figure 1 and Figure 2). Under low protein condition, octenidine dihydrochloride (OCT)-based product exhibited the highest efficacy, effectively eliminating all challenged bacteria within 15 s contact time. This is followed by polyhexamethylene biguanide (PHMB)-based product, achieving a 5-log reduction against *Staphylococcus aureus* at 60 s of contact time. In contrast, all five HOCl-based products only achieve a 5-log reduction after 2 min of contact time. The log reduction kinetics based on contact time is similar among all four HOCl-products except Antaviro^®^ (Figure 1). Antaviro^®^ is the only HOCl-based product to achieve a 5-log reduction after 15 s contact time. 

### 2.2. Efficacy of Wound Irrigation Solutions under High Protein Condition

It is known the high protein concentration can affect the antimicrobial efficacy of actives or molecules; therefore, a comparison under high protein conditions becomes necessary to stringently evaluate the bactericidal activities of wound products. 

The antimicrobial efficacy of OCT is not affected under high protein condition, achieving a bacterial reduction of more than 5-log within 15 s of contact time (Figure 2). Comparatively, the efficacy of PHMB decreased slightly, but it still achieved a 5-log reduction at 2 min of contact time. In contrast, none of the HOCl-based products were able to achieve more than a 3.5-log reduction even after 30 min of contact time. Interestingly, Antaviro^®^, which has the shortest contact time among the HOCl-based products under low protein conditions, now exhibited the lowest antimicrobial efficacy (a mean log reduction of 1.61 after 30 min of contact with the bacteria) compared to other HOCl-based products (Figure 2).

## 3. Materials and Method

### 3.1. Antimicrobial Solutions

In this study, the antimicrobial efficacies of seven commercially available wound irrigation solutions, each featuring varying active agent compositions, were assessed. Out of these, five solutions were formulated with HOCl, one with OCT, and another with PHMB. Established antimicrobial products, such as OCT- and PHMB-based wound irrigation solutions with known antimicrobial activities as demonstrated by EN 13727 were included as a ‘positive control’ [12,13]. The 0.85% NaCl was included as a ‘negative control’. A detailed summary of the products and their respective active substances can be found in Table 1. 

### 3.2. Challenge Conditions 

The antimicrobial test solutions were assessed in accordance with the EN 13727:2012+A2:2015 standard, under two stipulated conditions—clean and dirty. The ‘low protein’ (clean) condition involved the inclusion of 0.3% bovine serum albumin as an interfering substance, while the ‘high protein’ (dirty) condition incorporated 3% bovine serum albumin and an additional 3% sheep erythrocytes (HemoStat Laboratories, Dixon, CA, USA) as interfering substances to simulate the blood commonly encountered on skin/wound surfaces. 

### 3.3. Test Suspension

Bactericidal activity was assessed using *Staphylococcus aureus* ATCC 6538 (ATCC, Manassas, VA, USA) as the test microorganism. The working culture was prepared in adherence to the EN 13727:2012+A2:2015 standard protocol. Under the low protein condition, the bacterial suspension was prepared in a diluent solution containing 1 g/L Tryptone and 8.5 g/L NaCl. In contrast, for the high protein condition, the bacterial suspension was prepared in a tryptone soy broth (30 g/L, Oxoid CM0129B, Oxoid Ltd., Hampshire, UK), as described by Severing and colleagues (2022) [12]. The bacterial suspension was subsequently adjusted to a range between 1.5 × 10^8^ cfu/mL and 5.0 × 10^8^ cfu/mL using a spectrophotometer (Biobase Group, Shandong, China). The bacterial suspension was serial diluted up to 10^−7^. Subsequently, 1 mL of both the 10^−6^ and 10^−7^ bacterial suspensions (both low and high protein suspensions) were plated in duplicate using the pour plate method on tryptone soy agar (40 g/L, Oxoid CM0131B, Oxoid Ltd., Hampshire, UK). 

### 3.4. Quantitative Suspension Test and Challenge Conditions

Quantitative suspension tests were performed according to the dilution-neutralization method outlined in EN 13727:2012+A2:2015. In brief, 1 mL of the interfering substance was transferred into a sterile tube containing 1 mL of bacterial suspension and mixed for 2 min. After 2 min, 8 mL of test solution was added, and it was incubated in a cooler incubator at 20 °C for the specified contact time. At the end of each contact time (15 s, 30 s, 60 s, 2 min, 5 min, 15 min, and 30 min), 1 mL of the mixture was transferred to a tube containing 8 mL of the appropriate neutralizer and 1 mL of distilled water. The concentration of bacteria in this mixture is expected to be in the range between 1.5 × 10^6^ cfu/mL and 5.0 × 10^6^ cfu/mL. After a neutralization time of 10 s, 1 mL of the neutralized test mixture and serial dilutions (ranging from 10^−1^ to 10^−5^) were plated in duplicate and inoculated using the pour plate technique. Colonies were counted after incubation for 24–48 h at 37 °C. Bacterial reduction was calculated as the viable colonies before exposure to a disinfectant minus the viable colonies after exposure. Measurements were as follows: Lg R = lg N_0_ − lg Na, where N_0_ is the number of cfu/mL in the test mixture at the beginning of the contact time, and Na is the number of cfu/mL in the test mixture at the end of the contact time and before neutralization. Additionally, test validations (control A, control B, and control C) were conducted in accordance with the guidelines outlined in the EN 13727:2012+A2:2015 standards. Control C was performed to validate the dilution-neutralization method and ensure that the neutralizer used can neutralize the antimicrobial activity of products. A total of 1 mL of interfering substance was mixed with 1 mL of diluent. This is followed by the addition of 8 mL of test product to the tube. At the end of the contact time, 1 mL of this mixture was transferred into a tube containing 8 mL of neutralizer. After 5 min, 1 mL of validation suspension (3.0 × 10^2^ to 1.6 × 10^3^ cfu/mL) was added, and the tube was placed in a cooler incubator at 20 °C for 30 min. At the end of this time, a 1 mL sample of the test mixture C was taken and inoculated using the pour plate technique. The colony count of Control C must be equal to or greater than 50% of the 1/10 dilution of the validation suspension. The result for Control C is presented in Supplementary Tables S1 and S2. The neutralizers used for each test solution are summarized in Table 2. 

## 4. Discussion 

Antimicrobials, such as antiseptic-based products, continue to play an important role when it comes to managing localized bacterial infestation in wounds, especially against multi-drug resistant microorganisms [2,21]. However, their activity can be hampered by the presence of proteins to lower their bactericidal efficacy resulting in a longer contact time [11,12,13]. Together with previous studies, our study has further proven that this protein interference on antimicrobial activities does not apply to all actives. Consistent across different studies, OCT-based products such as octenilin^®^, are minimally affected by proteins, achieving a 5-log reduction in the shortest assessed contact time of 15 s [11,13]. This, however, was not the case for PHMB-based products, with varying antimicrobial efficacies when challenged against ‘dirty’ condition stimulated by wound exudate in different studies [11,13]. Using chronic wound exudates, 0.1% PHMB-based product has been shown to exhibit a lower antimicrobial efficacy, attaining a 5-log reduction only after 15 min contact time [11,13]. The mixed population of microbiota in the wound exudate has been suggested as a factor that can affect antimicrobial efficacy of the tested antiseptics [13]. Despite wound exudate being a challenge material of high relevance, unknown variations can exist in the preparations used in the different in vitro studies. For example, it has been demonstrated that different EN 13727 studies can yield varying antimicrobial results (e.g., 0.04% PHMB), even when similar wound exudate from chronic wounds was employed [11,12,13]. The variability associated with the use of wound exudate demonstrates the need for a simple, standardized testing condition, such as those used in this study for reproducibility.

As part of their mode-of-action, both cationic molecules, OCT and PHMB attach to the negative charged bacterial membrane to initiate bacterial elimination [22,23,24,25]. OCT in particular, have recently been shown to act in a cascading manner by first, neutralize surface charges of gram-positive and gram-negative bacteria, followed by non-specific disruption of cell membrane leading to permeabilization and content leakage of the bacterial cell [24,25]. Based on the antimicrobial efficacies of both OCT and PHMB, this binding specificity towards bacterial cell membrane does not seem to be significantly sequestered in the presence of high protein, suggesting minimal direct molecular interaction between the antiseptic molecule and the protein contaminants per se [11,12,13]. In contrast, HOCl is heavily affected by protein interference. According to Severing and colleagues (2022), the likely interaction of the highly reactive chlorine-based species with the exposed amino acids of the peptides can possibly lead to the exhaustion of these chlorine actives, reducing the freely available chlorine species for bacteria killing [12].

The antimicrobial efficacy of HOCl-based products has been reported to be dependent on the concentration of the NaOCl/HOCl, with products containing <0.08% NaOCl losing their antimicrobial efficacy against both gram-positive and gram-negative bacteria in high protein environments [15]. Therefore, the lower chlorine concentration (<0.005% to 0.02% HOCl) in all the five tested HOCl-based products could also be the attributing factor to the lower antimicrobial activities in the high protein environment, observed in this study. Not only does high protein environment affect the antimicrobial activities of the HOCl-based products, the presence of a complex bacterial aggregate such as biofilm can also render the loss of antimicrobial efficacy of HOCl against biofilm [14,26,27,28]. The pervasive nature of biofilm in chronic wounds is often an important consideration when choosing the correct products for wound bed preparation. Given the recent report by Rembe and colleagues (2020), in which HOCl with lower chlorine concentration were found to be significantly less effective against biofilm compared to either OCT or PHMB-based wound irrigation solutions, it can be postulated that the efficacies of the five HOCl-based products against biofilm will likely be low [14].

To date, there is no universal method/standard for evaluating antimicrobial products for wound care. One of the more commonly used testing methodologies being employed by manufacturers is EN 13727. However, there is a limitation when using the standard testing condition of EN 13727 to test wound antiseptic products—the standard testing condition, especially in low protein condition (0.3 g/L bovine serum albumin) does not mimic the environment encountered in the clinical application of products in wound care, resulting in potential bias and ‘overestimation’ of the antimicrobial efficacy of the tested products. As clinicians often rely on the efficacy profiles of antimicrobial products for product selection, this bias can potentially lead to the selection of a suboptimal product, and the adherence to an inaccurate contact time for bactericidal effect in the wound. For example, the contact time of Granudacyn^®^ to achieve a 5-log bacterial reduction would have been deemed to be 2 min from the results in the low protein condition. However, under the high protein environment, Granudacyn^®^ only achieved approximately a 2-log reduction in the same contact period. The latter is consistent with published studies of the EN 13727 testing of Granudacyn^®^ in different protein environments [12,15]. Interestingly, most of the manufacturer’s suggested contact time for wounds/encrustations for the various HOCl-based products, such as Dermacyn^®^, Hydrocyn^®^ Aqua, and Granudacyn^®^ is relatively short—aligning to the contact time in a low, rather than high protein conditions (Figure 1; Table 1). Based on our findings, a longer contact time beyond 30 min would be needed for all the five tested HOCl-based products to achieve the desirable antimicrobial efficacy of 5-log bacterial reduction, when high soiling is present. However, the practicality of this in the real-world wound management environment is questionable when patient’s turnaround time is considered.

The limitations of this study that warrant to be discussed are as follows: first, only a single representative gram-positive bacterium, *S. aureus* was employed for the investigation, making the translation of these results to gram-negative bacteria not possible. Despite this limitation, results from Severing and colleagues (2022) using the same testing conditions as this study on HOCl-based solutions has shown comparable antimicrobial results between both gram-positive and gram-negative bacteria [12]. Second, the reason(s) underlying the differences in the antimicrobial efficacies between the HOCl-based solutions (i.e., pH, chlorine content, oxidation-reduction potential etc.) have not been fully elucidated. Although out of the scope of this study, the investigation of this remains a topic of interest as it will provide further understanding to improve the HOCl-based solutions in wound care. 

## 5. Conclusions

In summary, the assessment of the five commercially available HOCl-based wound irrigation solutions using EN 13727, demonstrates that HOCl-based products are significantly affected by protein interference, resulting in a lowered bactericidal activity in a high protein environment compared to OCT- and PHMB-based wound irrigation solution. The findings from this study warrant the testing of different wound antiseptic products in a more ‘realistic’ conditions of higher protein content (3 g/dL) for a better assessment of the antimicrobial efficacy for wound care. 

## Figures and Tables

**Table 1:** Summary of the wound irrigation products used in this study.

Product	Manufacturer	Actives	Suggested Contact Time
octenilin^®^ wound irrigation solution	Schülke & Mayr GmbH, Norderstedt, Germany	0.05% OCT	Not indicated
Prontosan^®^ Wound Irrigation Solution	B. Braun Medical AG, Sempach, Switzerland	0.1% PHMB	15 min—for wound covering a large area and also wounds difficult to access [17]
Dermacyn^®^ wound care solution	Sonoma pharmaceuticals, Woodstock, GA, USA	99.97% oxidized water, 0.004% NaOCl, 0.003% HOCl, 0.023% NaCl	10 min on affected area [18]
Hydrocyn^®^ Aqua	Vigilenz Medical Devices Sdn. Bhd, Bukit Mertajam, Malaysia	0.003% HOCl, 0.1% NaOCl and NaCl	15 min on wound [19]
Granudacyn^®^ wound irrigation solution	Mölnlycke Health Care AB, Gothenburg, Sweden	<0.005% NaOCl and HOCl	60 s for any encrustations; 15 min to remove stubborn encrustations [20]
Electrocyn™ Soma advanced wound irrigation solution	V3bio Sdn. Bhd., Simpang Ampat, Malaysia	≤0.1% HOCl, NaCl and NaOCl	Not indicated
Antaviro^®^	Ionic Global Sdn. Bhd., Perai, Malaysia	0.02% HOCl, <0.01% NaCl	Not indicated
0.85% NaCl	Merck KGaA, Darmstadt, Germany	0.85% NaCl	-

OCT: octenidine dihydrochloride; PHMB: polyhexamethylene biguanide; NaOCl: sodium hypochlorite; HOCl: hypochlorous acid; NaCl: sodium chloride.

**Table 2:** Neutralizers used to counteract the antibacterial activity of the test product.

Test Product	Formulation of Neutralizers
octenilin^®^ wound irrigation solution	60 g/L Tween 80, 8 g/L Sodium dodecyl sulphate, 6 g/L Lecithin
Prontosan^®^ Wound Irrigation Solution	30 g/L Tween 80, 6 g/L Lecithin, 60 g/L Saponin, 1.0 g/L Histidine, 1.0 g/L Tryptone and 8.5 g/L NaCl
Antaviro^®^	
Dermacyn^®^ wound care solution	30 g/L Tween 80, 30 g/L Saponin, 10 g/L Sodium dodecyl sulphate, 3 g/L Sodium thiosulphate and 3 g/L Lecithin
Hydrocyn^®^ Aqua	
Granudacyn^®^ wound irrigation solution	
Electrocyn Soma™ advanced wound irrigation solution	
0.85% NaCl	

## Data Availability

Data are contained within the article and Supplementary Materials.

## Supplementary Materials

The following supporting information can be downloaded at: https://www.mdpi.com/article/10.3390/antibiotics13040309/s1, Table S1: Result of dilution-neutralization validation (Control C): Replicate 1. Table S2: Result of the dilution-neutralization validation (Control C): Replicate 2.
