# Supplementary material for: Impact of Interfering Substances on the Bactericidal Efficacy of Different Commercially Available Hypochlorous Acid-Based Wound Irrigation Solutions Commonly Found in South-East Asia
Source: Antibiotics (Basel). 2024 Mar 28;13(4):309. doi: 10.3390/antibiotics13040309 (PMC11047473; doi:10.3390/antibiotics13040309)
Supplement: Supplementary file 1 [file antibiotics-13-00309-s001.zip › antibiotics-2888339-supplementary.pdf]

antibiotics

MDPI

Supplementary data

Table S1. Result of dilution-neutralization validation (Control C): Replicate 1

CFU of validation suspension	Test Condition	Test Product	CFU of Control C
Nv/10: 141	Low protein condition	octenilin® wound irrigation solution	130.0
		Prontosan® Wound Irrigation Solution	137.5
		Antaviro®	135.0
		Dermacyn® wound care solution	132.0
		Hydrocyn® Aqua	140.0
		Granudacyn® wound irrigation solution	129.5
		Electrocyn Soma™ advanced wound irrigation solution	133.0
		0.85% NaCl	141.0
Nv/10: 131.0	High protein condition	octenilin® wound irrigation solution	122.0
		Prontosan® Wound Irrigation Solution	130.0
		Antaviro®	131.5
		Dermacyn® wound care solution	120.5
		Hydrocyn® Aqua	134.0
		Granudacyn® wound irrigation solution	143.0
		Electrocyn Soma™ advanced wound irrigation solution	146.0
		0.85% NaCl	124.0

Table S2. Result of the dilution-neutralization validation (Control C): Replicate 2

CFU of validation suspension	Test Condition	Test Product	CFU of Control C
Nv/10: 139.0	Low protein condition	octenilin® wound irrigation solution	137.5
		Prontosan® Wound Irrigation Solution	139.0
		Antaviro®	138.5
		Dermacyn® wound care solution	145.5
		Hydrocyn® Aqua	133.5
		Granudacyn® wound irrigation solution	142.5
		Electrocyn Soma™ advanced wound irrigation solution	137.0
		0.85% NaCl	132.5
Nv/10: 136.5	High protein condition	octenilin® wound irrigation solution	127.5
		Prontosan® wound Irrigation Solution	125.0
		Antaviro®	125.0
		Dermacyn® wound care solution	132.0
		Hydrocyn® Aqua	128.5
		Granudacyn® wound irrigation solution	127.5
		Electrocyn Soma™ advanced wound irrigation solution	130.0
		0.85% NaCl	130.0
